# Child linear growth trajectories during the first three years of life in relation to infant iron status: a prospective cohort study in rural Vietnam

**DOI:** 10.1186/s40795-022-00505-y

**Published:** 2022-02-15

**Authors:** Sarah Hanieh, Sabine Braat, Thach D. Tran, Tran T. Ha, Julie A. Simpson, Tran Tuan, Jane Fisher, Beverley-Ann Biggs

**Affiliations:** 1 Department of Infectious Diseases at the Peter Doherty Institute, University of Melbourne, Melbourne, VIC 3050 Australia; 2 Centre for Epidemiology and Biostatistics, Melbourne School of Population and Global Health, University of Melbourne, Victoria, 3010 Australia; 3 Global and Women’s Health, School of Public Health and Preventive Medicine, Monash University, Melbourne, VIC 3004 Australia; 4 Research and Training Centre for Community Development, Hanoi, Vietnam; 5 The Victorian Infectious Diseases Service, Royal Melbourne Hospital, Parkville, VIC 3052 Australia

**Keywords:** Iron, Child, Vietnam, Child growth, Iron status

## Abstract

**Background:**

Early childhood growth patterns have long-term consequences for health and disease. Little is known about the interplay between growth and iron status during childhood. We explored the interplay between linear growth and iron status during early childhood, by assessing child growth trajectories between 6 and 36 months (m) of age in relation to infant iron status at 6 months of age.

**Methods:**

A cohort study of infants born to women who had previously participated in a cluster randomized controlled trial of antenatal micronutrient supplementation, conducted in rural Vietnam. The relationship between child linear growth trajectories and infant iron status (ferritin concentration) was examined using latent growth curve modeling. Primary outcomes were height for age z scores (HAZ) and growth trajectory between 6 and 36 m of age.

**Results:**

A total of 1112 infants were included in the study. Mean [SD] HAZ scores decreased over time from –0·58 [0·94] at 6 m, to –0·97 [0·99] at 18 m, to –1·14 [0·89] at 36 m of age. There was a steep linear decline in the HAZ scores between 6 and 18 m of age, followed by a slower linear decline from 18 to 36 m of age. Ferritin concentration at 6 m of age was inversely associated with HAZ score at 6 m of age (-0·145, 95% CI [-0.189, -0.101]). There was no association between infant ferritin at 6 m of age and child growth trajectory between 6 and 36 m of age.

**Conclusions:**

Iron status at six months of age did not influence a child’s later linear growth trajectory in this cohort of rural Vietnamese children. Longitudinal studies with repeated ferritin and height measurements are required to better delineate this relationship and inform public health interventions.

**Supplementary Information:**

The online version contains supplementary material available at 10.1186/s40795-022-00505-y.

## Background

Understanding the determinants of linear growth during the first 1000 days of life is critical to inform interventions to prevent growth impairment and long term health effects associated with stunting (HAZ < –2SD) [1]. Iron is considered an essential micronutrient for growth and development, and young children are at risk of depletion during periods of rapid growth [2].The majority of term infants are born with adequate iron reserves that last for about the first 6 months (m) of life, after which a diverse diet with a variety of iron-rich foods is necessary to maintain robust iron stores [3].

Consequences of iron deficiency and anemia include: prematurity and low birth weight, impaired physical growth and, there is also strong evidence that infants with anemia are at risk for poorer cognitive and motor developmental outcomes [4], however limited evidence exists about the relationship between iron status (measured by ferritin concentration) and child growth in populations not receiving iron supplementation, and available findings are inconsistent [4–9]. This relationship is influenced by the complexity of iron metabolism in the setting of infection, the absence of reliable markers for iron status, and the wide range of determinants of growth making studies of these interactions difficult.

To better understand the interaction between iron status and growth in early childhood, we conducted post-hoc analysis of data from a cohort study in a rural province of Vietnam that followed children from birth to 36 m of age. Our primary aim was to identify whether infant ferritin concentration at 6m of age was predictive of the subsequent growth trajectory to 36m of age. We postulated that iron status at 6m of age would be a limiting factor in the subsequent growth trajectory of a child.

## Materials and methods

### Setting

The study was carried out in Ha Nam province, located in North Vietnam. Ha Nam has a population of approximately 799,400 people as of 2014, with most residents still working in subsistence agriculture. No antenatal or child iron supplementation programs were in place at the time of the study.

### Study Design and participants

A cohort study of infants born to women who had previously participated in a cluster randomized controlled trial (c-RCT) of antenatal micronutrient supplementation from enrolment (mean 12 weeks until 3 months postpartum) [5]. Women enrolled in the original c-RCT and their infants (ACTNR 12,610,000,944,033) were followed until 6 m post-partum in the original c-RCT (between September 2010 and Jan 2012) [5]. Women were randomised at the cluster (comune level) and received either (1) one tablet of iron-folic acid (IFA) taken daily (60 mg elemental iron /0·4 mg folic acid per tablet, 7 tablets per week); or (2) one capsule of IFA taken twice a week (60 mg elemental iron /1·5 mg folic acid per capsule; 2 capsules per week); or (3) one capsule of multiple micronutrients (MMN) taken twice a week (60 mg elemental iron, 1·5 mg folic acid, plus other micronutrients) [6]. The primary aim of the c-RCT was to compare the effect of twice weekly provision of antenatal IFA supplementation (either alone or in combination with other micronutrients) with daily provision of IFA supplementation, on maternal and infant outcomes during the first 6 months of life. Detailed information on the methodology used, including a table describing the composition of the supplements, has been previously published [5].

The cohort study followed the c-RCT described above. All 1171 infants live-born to women who were enrolled in the c-RCT and still alive at 6 m of age were eligible for enrolment in the cohort study. Children in the cohort study were followed from 6 months until 36 m of age (between May 2012 and May 2014). Written informed consent was obtained from carers of the children. The study protocol was approved by the Melbourne Health Human Research Ethics Committee and the Ha Nam Provincial Human Research Ethics Committee. Children were seen at the commune health station at 6, 12, 18, 24, 30 and 3 m of age.

### Study measurements

#### Anthropometric measurements

Infant height/length (cm) was measured at 6-monthly intervals between 6 and 36 m of age using a portable Shorr Board (Shorr Productions) during a study-related visit to the clinic. Research staff recorded triplicate measurements of anthropometric measures, a second observer checked all measurements, and the median measurement was obtained. HAZ scores were calculated based on the child’s age and median measurement of height, using WHO anthro [7]. For participants less than 24 months recumbent length was measured, and for participants 24 months and over standing height was measured. Mild, moderate, severe stunting was defined as HAZ scores less than one, two, and three standard deviations below WHO growth standards, respectively.

#### Iron measurements

One ml of venous blood was collected for serum ferritin concentration at 6m of age during a study-related visit to the clinic. Samples were frozen at –20 °C and transported to the Alfred Hospital, Melbourne, Australia for testing. Serum ferritin (ug/L) was analysed using a chemiluminescent microparticle assay (Architect ci16200; Abbott Diagnostics). Infant iron deficiency was defined as serum ferritin less than 12ug/L [8]. Infant haemoglobin (g/L) was measured using HemoCue (HemoCue AB, Angelholm, Sweden) during the same study-related visits. Infant anaemia was defined as haemoglobin less than 110 g/L [9].

#### Demographic measurements

Maternal sociodemographic factors were assessed using a standardized questionnaire administered by trained research staff at enrolment as part of c-RCT. The questionnaire included information on demographics, maternal age (years), maternal occupation, education (primary school, secondary school, university/college), and pregnancy history.

### Statistical methods

The sample size was determined by the available data on the infants of mothers participating in the c-RCT. The analysis sample included all children with at least one valid HAZ score between 6 and 36 m of age. HAZ scores were considered valid if the data point was collected within ± 2·5m of the planned 6-monthly visit; data outside this range were discarded. Categorical data are presented as percentages with frequency, and continuous data as mean and standard deviation (SD) or median and 25^th^–75^th^ percentile {Q1-Q3}, where appropriate.

Latent growth curve (LGC) modelling, which uses observed and latent variables, was used. Firstly, the trajectory of growth in HAZ scores over 6–36m was modelled unconditionally (without covariates) to identify its shape. The variance of the residual errors were initially independent and constrained to be equal over time. These assumptions of uncorrelated and homogeneous error variances over time were explored. The underlying assumption of multivariate normality of HAZ scores was justified by definition with natural reference being zero representing the average value of the reference population [7]. Secondly, an unadjusted conditional LGC model was used to evaluate the (linear) association of infant ferritin level at 6m (log base 2 transformed) and the latent growth parameters based on HAZ scores over 6–36m. Maternal iron intervention and clinically identified covariates (i.e., infant sex [female vs. male], maternal education [secondary school vs. primary school; university/college vs. primary school], birth weight (kg) [continuous], and child anaemia [yes vs no]) were added to obtain adjusted results.

The full information maximum likelihood (FIML) was chosen to obtain estimates of the model parameters and standard errors. The FIML handles missing data by the model and assumes missing data is at most missing at random. Nested models were compared using the likelihood ratio test (χ2) while non-nested models used the Akaike Information Criterion (AIC). The fit of the models was measured using the comparative fit index (CFI) and Tucker-Lewis Index (TLI) comparing the fitted model with a baseline model and root mean squared error of approximation (RMSEA) that penalises for excessive complexity. To determine goodness of the model fit, the following three fit indices were used: a CFI close to one (good fit > 0·95; adequate fit > 0·90), TLI close to one (> 0·95) and RMSEA (good fit < 0·05, adequate fit [0·05, 0·08], poor fit > 0·1) [10]. Data were analysed using Stata/IC Version 14 for Windows (StataCorp, 2015, College Station, TX, USA).

## Results

### Participants and characteristics

**Fig. 1:**
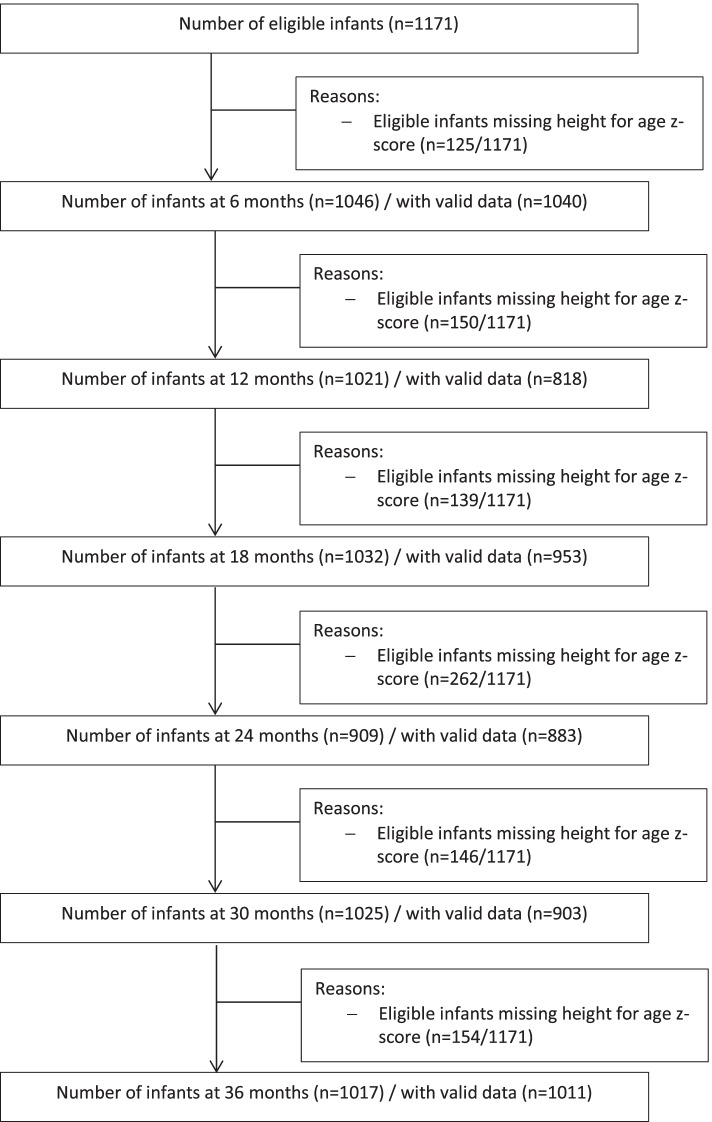
Flow chart of the infant/child’s participation during 30-months of follow-up. Height for age z-score is considered valid if the data is collected within ± 2·5m visit window

A total of 1171 live-born infants from the original c-RCT were enrolled in the follow-up cohort study. Three infants died between live-birth and 3-years of age, one due to diarrhoea and pneumonia and two children drowned. At any point in time between 6 and 36m, a total of 1112 (95·0%) children provided non-missing HAZ scores valid for inclusion in the analysis of whom 87·3% contributed three or more measurements. Of the infants with non-missing HAZ scores, valid HAZ scores (i.e., data collected inside  ± 2·5 m visit window) were available for 99·4%, 80·1%, 92·3%, 97·1%, 88·1%, and 99·4% at 6–36m respectively. A flow chart is presented in Fig. 1; online only. Infant and maternal demographic, biochemical, and anthropometric measurements are presented in Table 1 and 2. At 6m, mean (SD) haemoglobin was 110·3 (11·3) g/L and anaemia was present in 50·4% of infants. Median {Q1-Q3} ferritin was 31 {17–53} ug/L, with iron deficiency found in 15·5% of infants at 6m of age. On average, mothers were 26·6 years old at the start of the c-RCT and about two thirds were secondary school educated (66·8%). The distribution of the household wealth index was negatively skewed whereby the difference in means in adjacent quintiles was larger between the poorest and poor quintile compared to any other adjacent quintiles. The percentage of children with moderate stunting increased over time from 6·3% at 6m, 14·5% at 18m, to 16·8% at 36m of age.

**Table 1:** Infant and maternal characteristics of participants with at least one non-missing valid HAZ score during 6–36 months (*N* = 1112)

**Infant and maternal characteristics**	
**Infant characteristics at birth**	**Values**
Demographic factors	
Birth weight (grams)^a^	3158 [399]
Gestational age (weeks)^b^	39.1 [2.0]
Preterm delivery (< = 37 weeks)^b^	127 (11.9%)
Male sex	590 (53·2%)
**Infant characteristics at 6 months of age**^f^	
Biochemical factors	
Haemoglobin (g/L)^c^	110·3 [11·3]
Anaemia (Hb < 110 g/L)^c^	512 (50·4%)
Ferritin (ug/L)^d^	31 {17–53}
Iron deficiency (Ferritin < 12ug/L)	143 (15·5%)
Iron deficient anaemia (Hb < 110 g/L, Ferritin < 12ug/L)	96 (10·4%)
**Maternal characteristics at child birth**	
Demographic factors	
Maternal age (years)^e^	26·6 [4·9]
Education	
Primary school	171 (15·4%)
Secondary school	742 (66·7%)
University/College	199 (17·9%)
Type of supplement taken during pregnancy in c-RCT	
Daily IFA supplements	375 (33·7%)
Twice weekly IFA supplements	382 (34·4%)
MMN supplements	354 (31·9%)

Values are mean [SD] or number (%), *HAZ* height-for-age z, *Hb* Haemoglobin, *c-RCT* cluster randomised controlled trial, *IFA* iron-folic acid, *MMN* multiple micronutrient

^a^Data on birthweight missing for 2 infants

^b^Data on gestational age missing for 46 infants

^c^Data on haemoglobin missing for 97 infants

^d^Data for ferritin missing for 190 infants

^e^Data on maternal age missing for 1 mother

^f^Characteristics of those infants with valid HAZ scores at 6 months

Height for age z-score is considered valid if the data is collected within +/- 2.5m window

**Table 2:** Anthropometric outcomes of participants with non-missing valid HAZ scores at each 6-monthly visit (*N* = 1112)

	Month 6	Month 12	Month 18	Month 24	Month 30	Month 36
	(*N* = 1040)	(*N* = 818)	(*N* = 953)	(*N* = 883)	(*N* = 903)	(*N* = 1011)
Weight (grams)	7705 [976]	9180 [1160]	10,246 [1238]	11,218 [1362]	12,302 [1480]^a^	13,096 [1560]
Length/ Height (cm)	66·0 [2.3]	74·5 [2.6]	79·6 [3.0]	84·0 [3.2]	88·2 [3.4]	91·1 [3.4]
Body Mass Index (kg/m^2^)	17.7 [1.6]	16.5 [1.4]	16.1 [1.2]	15.8 [1.2]	15.8 [1.2]	15.7 [1.2]
HAZ	–0·58 [0.94]	–0·68 [0.96]	–0·97 [0.99]	–1·03 [0.96]	–1·09 [0.92]	–1·14 [0.89]
Mild stunting (HAZ < –1)	321 (30.9%)	310 (37.9%)	483 (50.7%)	458 (51.9%)	498 (55.1%)	578 (57.2%)
Moderate stunting (HAZ <–2)	66 (6.3%)	64 (7.8%)	138 (14.5%)	136 (15.4%)	142 (15.7%)	171 (16.9%)
Severe stunting (HAZ <–3)	8 (0.8%)	5 (0.6%)	11(1.2%)	14 (1.6%)	10 (1.1%)	13 (1.3%)

Values are mean [SD] or number (%)

*HAZ* Height for age z-score

Height for age z-score is considered valid if the data is collected within +/-2.5m visit window

^a^Data on weight was missing for one child

### Child growth trajectory from 6–36 m of age

**Fig. 2:**
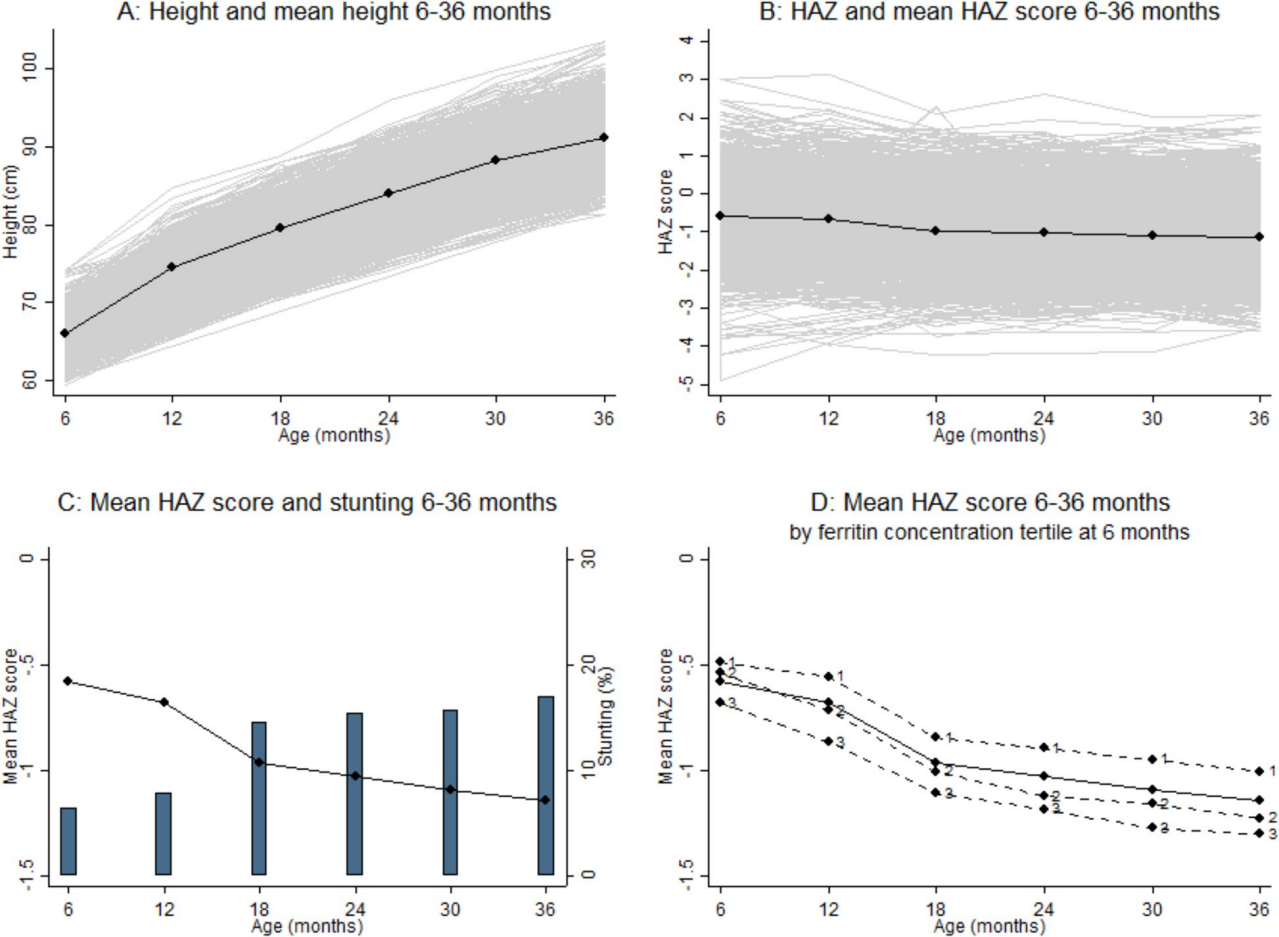
Individual trajectories, observed mean trend in HAZ scores and stunting prevalence between 6 and 36 months of age (*N* = 1112). **A** and **B** Solid black line represents the mean pattern 6–36 months. **B-D** HAZ = height-for-age z. **D** Ferritin concentration tertile at 6 months: 1 ( < 22 ug/L (low)), 2 ( [22, 44] ug/L (mid)), 3 (> 44 ug/L (high))

The average trajectory of height and HAZ scores over time and the magnitude of the individual variability around the mean trajectory is presented in Fig. 2, left and right panels respectively. Mean [SD] height increased over time from 66·0 [2·3] cm, to 79·6 [3·0] cm at 18 m, to 91·1 [3·4] cm at 36 m. Mean [SD] HAZ scores decreased over time from –0·58 [0·94] at 6 m, to –0·97 [0·99] at 18 m, to –1·14 [0·89] at 36 m of age. The average profile of the HAZ scores suggested a linear downward trend from 6 m with a change point at 18 m of age followed by a further linear downward trend up to 36 m of age. An unconditional piecewise linear LGC model was chosen to fit the trajectory of the six HAZ scores (6, 12, 18, 24 and 36 m) using three growth parameters: a latent intercept- describing the average HAZ score at 6 m (–0·538, 95% CI [–0·595, –0·482], *p* < 0·0001 with variance 0·760, 95% CI [0·685, 0·842], *p* < 0·0001); a latent slope- describing the average 6-monthly change in HAZ scores over time from 6 to 18 m (–0·228, 95% CI [–0·247, –0·228], *p* < 0·0001 with variance 0·042, 95% CI [0·033, 0·055], *p* < 0·0001); and a second less steep latent slope- describing the average 6-monthly incremental change in HAZ scores over time from 18 to 36 m of age (–0·056, 95% CI [–0·066, –0·046], *p* < 0·0001 with variance 0·016, 95% CI [0·013, 0·019], *p* < 0·0001). The correlation between the intercept and the slope 6 m to 18 m (–0·021, 95% CI [–0·142, 0·100], *p* = 0·74) and between both slopes (0·015, 95% CI [–0·127, 0·157], *p* = 0·83) was negligible, while the correlation between the intercept and slope from 18 to 36 m was negative (–0·424, 95% CI [–0·511, –0·337], *p* < 0·0001). The associated fixed factor loadings can be seen in Figure S1; online only, in the arrows pointing from the latent growth parameters to the observed monthly HAZ scores. Most fit indices indicated a good fit (RMSEA = 0·072; CFI = 0·991; TLI = 0·989).

### Association between child ferritin concentration at 6 m of age, and child growth trajectory between 6 and 36 m of age

We fitted the unadjusted LGC model as depicted in Figure S1 to explore the association of infant ferritin at 6m of age with the growth trajectory from 6- 36m of age. At 6m of age, (log base 2 transformed) ferritin was significantly inversely associated with HAZ scores (–0·066, 95% CI [–0·114, –0·018] *p* = 0·0067). Infant ferritin at 6m of age was not predictive of child growth trajectory between 6 and 36m of age.

Incorporating maternal c-RCT intervention female sex, birth weight, maternal education, and child anaemia with early infant iron in an adjusted LGC model by regressing on all three latent growth parameters, the adjusted association between (log_2_ transformed) ferritin and HAZ at 6m of age was stronger (–0·147, 95% CI [–0·191, –0·103] *p* < 0·0001) compared to the unadjusted association. Exploring the associations between the three latent growth parameters and each covariate suggested a significant association with sex (females had a higher HAZ at 6m and a steeper decrease HAZ at 18–36m compared to males), birth weight (infants with a higher than average birth weight had higher HAZ at 6m of age and a steeper decrease in HAZ at 12–18m and 24–36m, than those with an average birth weight), and maternal supplementation in the c-RCT (infants whose mother was randomised to twice weekly IFA had a lower HAZ at 6m than those on daily IFA), however there was no significant difference between trial arm and growth trajectory during the 12-18m or 18-36m periods. Most fit indices indicated a good fit for all models (Table 3).

**Table 3:** Parameters and model fit indices for Latent Growth Curve Models and ferritin at 6 months of age (*N* = 1112)

	Conditional LGC Including ferritin			Conditional LGC Adjusted including ferritin		
	Estimate	95% CI	*P*-value	Estimate	95% CI	*P*-value
**Growth parameters**						
**6 months**						
Intercept 6 months	–0·220	(–0·457, –0·018)	0·0700	–0·060	(–0·325, 0·206)	0·6588
**Ferritin 6 months**^a^	**–0·066**	**(–0·114, –0·018)**	**0·0067**	**–0·147**	**(–0·191, –0·103)**	**<0·0001**
Female sex	-	-	-	0·321	( 0·217, 0·424)	<0·0001
Birth weight (kg)^b^	-	-	-	1·079	( 0·947, 1·210)	<0·0001
Child anaemia at 6 months	-	-		0·039	(–0·067, 0·145)	0·4726
Maternal education (secondary school)^c^	-	-	-	0·118	(–0·024, 0·260)	0·1032
Maternal education (university college)^c^	-	-	-	0·245	( 0·070, 0·420)	0·0060
Maternal supplementation (twice IFA)§	-	-	-	–0·166	(–0·288, –0·044)	0·0075
Maternal supplementation (MMN)§	-	-	-	–0·023	(–0·147, 0·101)	0·7186
**6–18 months**						
Linear slope 6–18 months	–0·205	(–0·288, –0·122)	<0·0001	–0·226	(–0·329, –0·122)	<0·0001
**Ferritin 6 months**^a^	**–0·005**	**(–0·021, 0·012)**	**0·5793**	**–0·002**	**(–0·019, 0·015)**	**0·8037**
Female sex	-	-	-	–0·025	(–0·064, 0·015)	0·2280
Birth weight (kg)^b^	-	-	-	–0·073	(–0·124, –0·021)	0·0058
Child anaemia at 6 months	-	-	-	–0·029	(–0·069, 0·012)	0·1654
Maternal education (secondary school)^c^	-	-	-	0·025	(–0·030, 0·080)	0·3684
Maternal education (university college)^c^	-	-	-	0·134	( 0·066, 0·203)	0·0001
Maternal supplementation (twice IFA)§	-	-	-	0·009	(–0·038, 0·056)	0·7054
Maternal supplementation (MMN)§	-	-	-	–0·028	(–0·076, 0·019)	0·2465
**18–36 months**						
Linear slope 18–36 months	–0·038	(–0·080, –0·005)	0·0814	–0·011	(–0·063, 0·041)	0·6732
**Ferritin 6 months**^**a**^	**–0·004**	**(–0·012, 0·005)**	**0·3853**	** 0·005**	**(–0·003, 0·014)**	**0·2234**
Female sex	-	-	-	–0·071	(–0·091, –0·051)	<0·0001
Birth weight (kg)^b^	-	-	-	–0·076	(–0·101, –0·050)	<0·0001
Child anaemia at 6 months	-	-	-	–0·008	(–0·029, 0·012)	0·4314
Maternal education (secondary school)^c^	-	-	-	–0·024	(–0·051, 0·004)	0·0873
Maternal education (university college)^c^	-	-	-	–0·029	(–0·063, 0·004)	0·0892
Maternal supplementation (twice IFA)^d^	-	-	-	–0·016	(–0·039, 0·007)	0·1798
Maternal supplementation (MMN)^d^	-	-	-	–0·017	(–0·040, 0·007)	0·1702
**Variance-Covariance**^e^						
Variance Intercept 6 months	0·752	( 0·678, 0·834)	<0·0001	0·569	( 0·509, 0·635)	-
Variance Slope 6–18 months	0·042	( 0·033, 0·055)	<0·0001	0·041	( 0·031, 0·053)	-
Variance Slope 18–36 months	0·016	( 0·013, 0·019)	<0·0001	0·014	( 0·012, 0·017)	-
Correlation Intercept-Slope 6–18 months	–0·024	(–0·146, 0·097)	0·6989	0·000	(–0·020 0·020)	0·969
Correlation Intercept-Slope 18–36 months	–0·431	(–0·518, –0·344)	<0·0001	–0·032	(–0·041, –0·024)	<0·0001
Correlation Slope 6–18 and 18–36 months	0·015	(–0·127, 0·157)	0·8370	–0·000	(–0·004 0·003)	0·849
Variance Ferritin 6 months	1·654	( 1·509, 1·812)	<0·0001	1·655	( 1·511, 1·813)	-
**Fit indices**						
CFI	0·991			0·992		
TLI	0·988			0·986		
RMSEA (Prob[RMSEA≤0·05])	0·064 (0·0421)			0·041 (0·9468)		

*CI* Confidence Interval, *IFA* Iron-Folic Acid, *MMN* Multiple MicroNutrients, *CFI* Comparative Fit Index, *TLI* TuckerLewis Index, *RMSEA* Root-Mean Standardised Error of Approximation, *LGC* Latent Growth Curve

^a^Ferritin is (log_2_ -transformed) ferritin

^b^Birth weight is (mean-centered) birth weight (kg)

^c^Maternal education compared to primary school

^d^Maternal supplementation compared to daily iron-folic-acid

^e^Only the random effects related to the latent growth parameters and ferritin are presented

## Discussion

To our knowledge, this is the largest study to investigate linear growth trajectories in pre-school children in South East Asia and their relationship to infant iron status. Using latent growth curve analysis, we examined the relationship between iron status at 6 months of age and linear growth patterns between 6 and 36m of age. We found that i) iron status at 6 m of age was not a significant determinant of linear growth trajectory during the first 36 m of life; and ii) linear growth trajectories declined steeply from 6 until 18 m, with a slower linear downward trend up to 36 m of age; and iii) infant ferritin levels at 6 m of age negatively correlated with 6 m HAZ scores.

We found no relationship between infant ferritin at 6 m of age and growth trajectory between 6 and 36m of age. Previous studies assessing the role of iron on linear growth in children have demonstrated heterogeneous results [11–13]. Several studies have reported adverse effects with iron deficiency (including decreased linear growth). Soliman et al. [12]. found that 40 toddlers (aged less than 4 years of age) with iron deficiency anaemia were significantly shorter with markedly slower growth velocity at presentation compared to age- and sex-matched normal controls. These findings were reversible with iron therapy. In contrast, Thorsdottir et al. found that iron deficiency at 12 months was associated with faster growth from birth to 12 months of age [13].

Our results suggest that infant iron stores do not influence later growth trajectory in this population in rural Vietnam. It is possible that the relative importance of iron status as a determinant of growth varies between settings*,* and that infant iron status is not a major determinant of growth in this setting. Previous studies have shown that there is a relatively low prevalence of iron deficiency (13%) in young children in rural Vietnam [14]. We found that maternal body mass index, maternal weight gain during pregnancy and maternal vitamin D concentration were associated with postnatal growth outcomes in rural Vietnam at 6 months of age [15, 16].

Our finding that child growth declines steeply from 6 until 18m, with a slower linear downward trend to 36m of age is similar to other studies, and supports the concept that early child growth is primarily influenced by early life factors, and especially the intrauterine environment (e.g maternal pre-pregnancy body mass index, weight gain during pregnancy and micronutrient status during pregnancy) [17]. Accordingly, interventions that address diets and nutritional status of mothers during pregnancy, including both nutrition-specific (fortification of foods, micronutrient supplementation) and nutrition-sensitive interventions around access to food and nutrient availability (e.g., agriculture, sanitation and hygiene, infection control) are associated with improved child growth [18]. Although we did not address antenatal determinants in this study, we have previously shown that maternal BMI and weight gain during pregnancy positively predict height for age z scores at 6 months of age [15]. Growth patterns described in previous studies also show the strong influence of region, as well as environmental, socio-economic and intergenerational factors [19–22].

The period from birth to the age of three is a time of rapid growth and a child’s iron requirements increase during these high-growth periods. During the first six months of life, term infants rely on the iron reserves they are born with, however following this period, infants are at high risk of iron deficiency if iron requirements for growth are not met from dietary or other sources [23]. It is has previously been shown that infants who grow rapidly are at high risk of depletion of iron stores [24]. In our study, children with higher height for age z-scores at 6 months of age had lower iron stores, suggesting that rapid growth during the first six months of life had depleted iron stores. However relatively few children (15·5%) became iron deficient.

We found that females had a higher z-score at 6m, but a steeper decrease in z scores at 18–36m compared to males. These sex differences in growth patterns have been previously described [15], and may reflect differences in feeding patterns during the toddler period on the basis of gendered cultural perceptions [25], or divergent patterns of care for girl children (e.g. less access to overall nutrition, lower likelihood of seeking healthcare if they are sick) [26].

The relatively high prevalence of anaemia in infants in this population may be due to factors other than ferritin deficiency, such as haemoglobinopathies or inflammatory disorders/infection. We did not test for haemoglobinopathies or measure C-reactive protein in this population, however previous studies have shown that hemoglobinopathies are not highly prevalent in the Kinh population in Vietnam, and are more commonly seen in ethnic minority groups [27]. Ha Nam province is a malaria free province. Capillary measurement of haemoglobin (with the Hemocue Machine) may also not be an accurate measure of haemoglobin concentration, which may have implications for the estimated prevalence of anaemia in this population [28].

Strengths of our study include the large sample size, standardised height measurements and the use of latent growth curve modelling to determine the relationships between infant iron status and growth outcomes during the first 36 m of life. The limitations of the study are that we were only able to collect a suitable volume of blood on 88% of infants for ferritin analysis at 6 months of age, the lack of serum ferritin data between 6 and 36 months of age, we didn’t collect information on other potential causes of iron deficiency anaemia such as haemoglobinopathies which may also contribute to impaired growth in the child, and that information on infant and child diets were not collected or analysed.

We were unable to confirm the impact of growth trajectory during the first six months of life on ferritin status at 6m due to the high proportion of missing data at the birth and 6 week time points. Also, we used height for age z scores in this analysis. The use and interpretation of HAZ scores, as opposed to absolute height, has been subject to debate, as standard deviations of the reference distribution vary by age. However, these variations have been shown to occur mainly with catch up growth, after two years of age [29]. To our knowledge, this remains the largest cohort study to document the association of early life iron status and HAZ scores over time. From a public health perspective our study highlights the importance of enhancing nutritional status in early life to ensure optimal growth in childhood.

## Conclusions

Our findings suggest that early life iron status is not a major determinant of a child’s later linear growth trajectory in this cohort of rural Vietnamese children. However the relationship between a child’s growth and iron stores is complex and longitudinal studies of repeated ferritin and growth measurements are required to better delineate this relationship and inform public health interventions.

## Supplementary Information

**Additional file 1: Figure S1. **Latent growth curve models of the effect of iron and child growth between 6 and 36 months. Latent variables are presented as circles and observed variables as rectangles. The arrows indicate that the variable was used to predict. Path diagram of the hypothesized unadjusted model of the association between iron at 6 months and child growth.

## Data Availability

The datasets used and/or analysed during the current study are available from the corresponding author on reasonable request.

## Abbreviations

BMI: Body mass index
CI: Confidence Interval
CFI: Comparative Fit Index
c-RCT: Cluster randomized controlled trial
HAZ: Height for age z scores
Hb: Haemoglobin
IFA: Iron folic acid
LGC: Latent growth curve
MMN: Multiple micronutrients
M: Months
RMSEA: Root-Mean Standardised Error of Approximation
SD: Standard deviation
TLI: Tucker-Lewis Index
WHO: World Health Organisation
Q: Quintile
