# Supplementary figures and images for: Child linear growth trajectories during the first three years of life in relation to infant iron status: a prospective cohort study in rural Vietnam
Source: BMC Nutr. 2022 Feb 15;8:14. doi: 10.1186/s40795-022-00505-y (PMC8845254; doi:10.1186/s40795-022-00505-y)

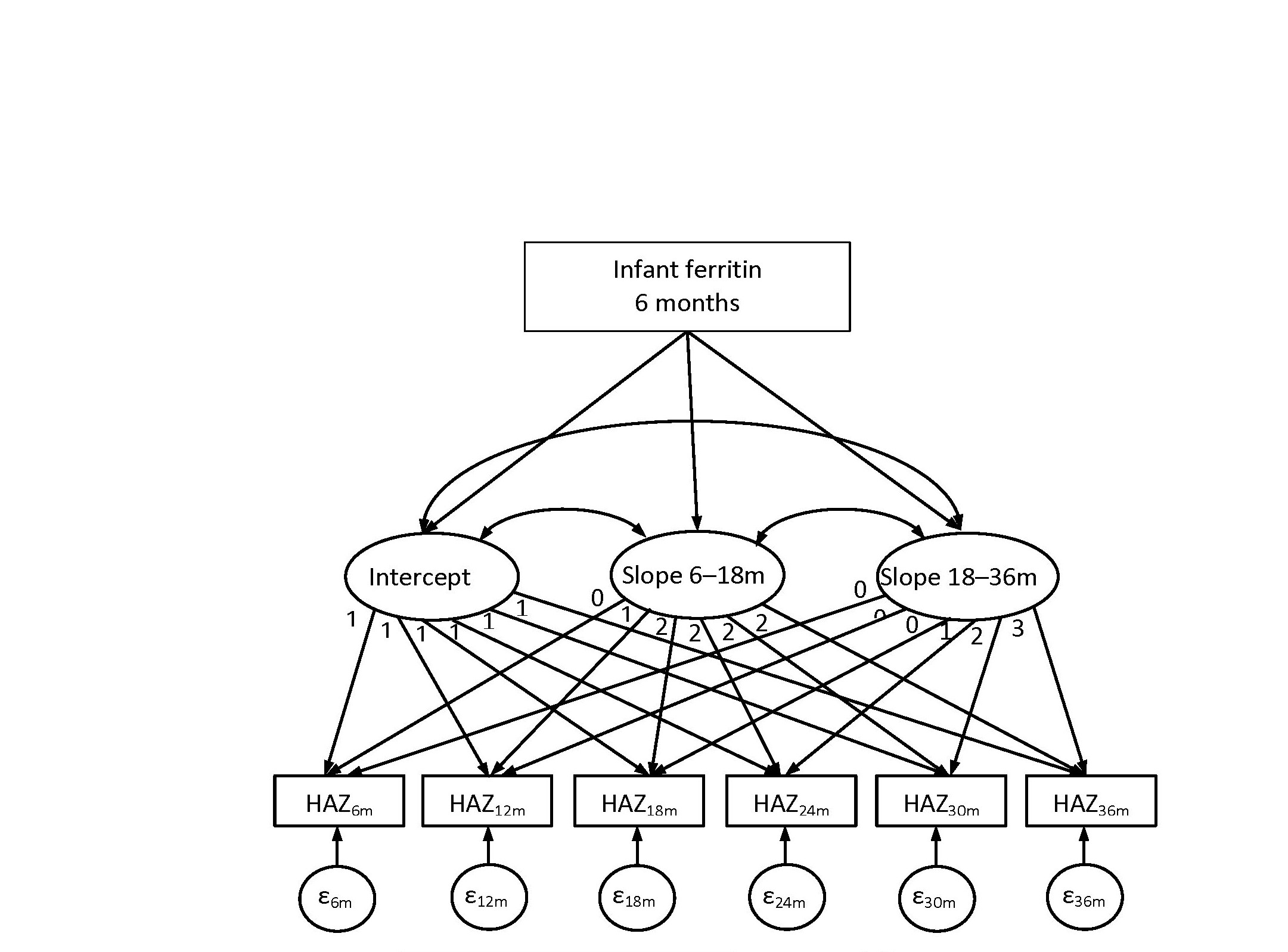

Supplement: Supplementary file 1 — Additional file 1: Figure S1. Latent growth curve models of the effect of iron and child growth between 6 and 36 months. Latent variables are presented as circles and observed variables as rectangles. The arrows indicate that the variable was used to predict. Path diagram of the hypothesized unadjusted model of the association between iron at 6 months and child growth. [file 40795_2022_505_MOESM1_ESM.jpg]
